# Spatio-temporal occurrence of *Culicoides* biting midges in the climatic regions of Switzerland, along with large scale species identification by MALDI-TOF mass spectrometry

**DOI:** 10.1186/1756-3305-5-246

**Published:** 2012-10-31

**Authors:** Christian Kaufmann, Irene C Steinmann, Daniel Hegglin, Francis Schaffner, Alexander Mathis

**Affiliations:** 1 Vector Entomology Unit, Institute of Parasitology, Vetsuisse Faculty, University of Zürich, Winterthurerstrasse 266a, Zürich, 8057, Switzerland

**Keywords:** Culicoides, Biting midge, Vectors, Obsoletus group, Pulicaris group, Species identification, MALDI-TOF MS, Monitoring, Abundance, Climatic region

## Abstract

**Background:**

*Culicoides* biting midges are incriminated as biological vectors of a number of viruses, e.g. bluetongue virus. In order to define vector-free periods/areas and to assess the vectorial role of the various *Culicoides* species, a comprehensive knowledge on their spatio-temporal occurrence is required.

**Methods:**

Biting midges were monitored on farm sites with livestock in the defined climatic regions, including high altitudes, of Switzerland by overnight trapping at 12 locations once a week over three years using UV-light traps. Based on morphological features, they were separated into three groups (i.e. Obsoletus, Pulicaris, other *Culicoides* spp.), and identification to the species level was achieved by protein profiling using MALDI-TOF mass spectrometry.

**Results:**

Around 550,000 biting midges in total were collected, revealing a dominance (82 to 99%) of the Obsoletus group species up to an altitude of 1,200 m and of the Pulicaris group species above 1,500 m (85% at the highest trapping site at 2,130 m). The maximum number of midges collected in a summer night (756 to 19,682) as well as the total number of midges caught over three years (from 6,933 to 149,439) varied highly among the sites, whereas the annual variation in total midge abundance at the locations was statistically insignificant. MALDI-TOF MS of 100 randomly selected individual biting midges per trapping site yielded high quality spectra for 1,187 of the 1,200 (98.9%) specimens of which 1,173 could be assigned to one of the 15 *Culicoides* species for which biomarker mass sets are available in the reference database.

**Conclusions:**

There are no biting midge-free zones in all of the agriculturally utilized areas (including alpine summer pastures) of Switzerland. Annual variations of midge numbers at the sampled locations were low, indicating that monitoring of midges should preferably be done by investigating a large number of sites for one season instead of few locations for extended periods of time. High throughput species identification of midges by MALDI-TOF MS is feasible, and this technique adds to other recently developed methods for the identification of midges (PCRs in various formats, interactive identification keys), facilitating epidemiological and biological in-depth studies of these important insects.

## Background

The unexpected and explosive outbreak of the bluetongue disease in Northern and Central Europe in 2006 triggered Europe-wide activities to monitor the biological vectors of the disease, biting midges of the genus *Culicoides* (Diptera, Ceratopogonidae), with the primary aim to define vector-free periods [1-5]. Thus, overviews of seasonally vector-free periods, determined for different years, are available for a number of countries [6].

*Culicoides* midges are incriminated as putative vectors of other orbiviruses of relevance for Europe at present (African horse sickness virus, epizootic haemorrhagic disease virus, Toggenburg virus; [7-11]). Very recently, an Orthobunya virus has emerged in Europe (‘Schmallenberg virus’), causing fever, diarrhoea, malformed new-borns and abortion [12], and this virus is assumed to be transmitted by biting midges and/or mosquitoes in analogy to the known vectors of related viruses [13]. Interestingly, biting midges of another Ceratopogonidae genus (*Forcipomyia*, subgenus *Lasiohelea*), have recently been incriminated as vectors of protozoan parasites of the genus *Leishmania* in Australia [14]. In addition to their emerging role as vectors, biting midges are a well-known nuisance pest in many parts of the world, and they can cause insect bite hypersensitivity (‘sweet itch’), particularly in equids, but the species that cause the clinical symptoms under field conditions are not known [15].

The identification of the tiny (1–3 mm) biting midges is primarily carried out using morphological features, and a recently developed interactive identification key will be of great practical value [16]. The wing patterns allow for a rapid classification of the insects into groups (Obsoletus group, Pulicaris group, other *Culicoides*[17]http://www.culicoides.net). Certain species can be identified with their wing pattern only, while others require time consuming microscopic analysis of slide-mounted insect preparations [18,19]. Therefore, morphological identification can be a time-consuming procedure and is known to be a very difficult task in many cases even for expert taxonomists [20], due to faint characteristics or intraspecific variability [21]. Furthermore, the existence of cryptic species, i.e. morphologically indistinguishable midges that are genetically distinct, has recently been described [21,22]. Hence, in large entomological surveys, trapped midges are for practical reasons first grossly separated to group level (Obsoletus, Pulicaris, other *Culicoides*) and further identified to species level depending on the available resources. Generally, species of the Obsoletus group dominated in European monitoring projects e.g. [2-5,23,24]. In a recent study in Austria, for example, a nation-wide monitoring at 54 locations at altitudes from 116 to 1,190 meters above sea level (m.a.s.l.) and run over 15 months revealed 90.2% of the biting midges as belonging to the Obsoletus group, 5.3% to the Pulicaris group, and 4.5% to other *Culicoides*[3].

Alternative approaches to midge identification include PCR. Several such assays in various formats have been developed for the specific detection of a number of *Culicoides* species [summarized in 22], including a microarray format for the identification of Obsoletus group species [25]. Recently, mass spectrometry (matrix-assisted laser desorption/ionization time of flight mass spectrometry, MALDI-TOF MS) has been evaluated as a diagnostic tool [26], and a reference database of biomarker masses covering 15 *Culicoides* species, including an abundant cryptic species, was established [27].

The aims of the present study were to determine over three years the spatio-temporal dynamics of biting midges in the defined climatic regions, including high altitudes, of Switzerland, and to apply MALDI-TOF MS for the species identification of a large number of field-collected specimens.

## Methods

### Trapping locations

The 12 trap locations were chosen in the vicinity of Swiss national basic climatological network reference stations (though no meteorological data were evaluated in the work presented) in 11 of the 12 defined climatic regions of the country [28] (Table 1). From the three climatic regions along the northern slope of the Alps (east, central, west), only two were considered (east, central). Instead, an additional second trap at high altitude (municipality ‘Juf’, 2,130 m) was run in the alpine region ‘North and Central Grisons’. The sites were distributed all over the country, covering low (270 m) and high (2,130 m) altitudes, areas with Atlantic climate and climate influenced by the Mediterranean Sea, and areas within and on both sites of the two mountain ranges (Alps, Jura).

**Table 1:** Features of the trapping locations and general entomological results of the 3 year monitoring

**Location (municipality) Coordinates**^**1 **^**(N/E)**	**Altitude**^**1**^	**Climate region**^**2 **^**(specification)**	**No. analysable trappings**^**3**^	**Total no. *Culicoides */ other insects**	**Overall *Culicoides *(%)**		
					**Obsoletus group**	**Pulicaris group**	**other *Culicoides***
Novazzano	270	South of the Alps^4^ (border to Italy)	97	19,307 / 132,004	88.5	1.8	9.7
45°50'30"/8°58'46"							
Dittingen	360	Eastern Jura mountain range (low altitude at northern slope)	109	149,439 / 113,677	98.6	1.0	0.4
47°26'31"/7°29'46"							
**Commugny**	**420**	**Western lowlands (shore of lake Geneva)**	**150**	**6,933 / 247,425**	**94.8**	**3.1**	**2.1**
** 46°19'20"/6°10'24"**							
Granges	500	Wallis (low altitude inner alpine valley with east-west orientation)	140	11,329 / 33,972	91.8	6.6	1.6
46°15'38"/7°27'52"							
**Wädenswil**	**620**	**North-eastern lowlands**	**145**	**64,358 / 144,627**	**94.4**	**4.0**	**1.6**
** 47°12'49"/8°39'53"**							
**Mühlethurnen**	**670**	**Central lowlands**	**146**	**18,070 / 155,267**	**96.4**	**2.2**	**1.4**
46°48'50"/7°30'41"							
Bennau	810	Northern slope of the Alps, central	112	24,009 / 164,150	91.4	7.2	1.4
47°8'55"/8°43'45"							
**Château d’Oex**	**940**	**Northern slope of the Alps, west**	**148**	**32,990 / 119,144**	**83.4**	**12.5**	**4.1**
** 46°28'27"/7°7'48"**							
**Chaumont**	**1,110**	**Western Jura mountain range (mountain top)**	**155**	**80,906 / 64,977**	**81.8**	**17.6**	**0.6**
** 47°1'38"/6°57'25"**							
Davos	1,560	North and Central Grisons (high altitude inner alpine valley with north-south orientation)	122	16,488 / 15,764	13.0	78.5	8.5
46°47'59"/9°49'51"							
Sils	1,800	Engadin (high altitude inner alpine valley with east-west orientation)	95	57,858 / 19,531	16.3	65.9	17.8
46°25'48"/9°45'48"							
**Juf**	**2,130**	**North and central Grisons (highest permanently inhabited village in Europe)**	**155**	**65,715 / 68,564**	**2.0**	**85.8**	**12.2**
** 46°26'43"/9°34'45"**							

Coordinates (latitude north/ longitude east) and altitudes (meters above sea level) according to Google Earth. Locations are listed in ascending order with regard to altitude. The six trapping locations with high sampling reliability (≥ 145 weekly catches) are in bold.

^2^ As defined and described [28].

^3^ Onderstepoort blacklight suction traps operated once per week, from approximately 2 h before dusk and until after dawn, for 3 years (no. = weeks, maximum number of trappings = 156).

^4^ Climate influenced by the Mediterranean Sea.

### Insect collection

Biting midges were caught on farms using the Onderstepoort blacklight suction traps [29] operated once per week, from approximately 2 h before dusk and until after dawn [17]. Criteria applied for the placement of the traps [17] were 1) presence of more than 10 head of ruminant livestock, i.e. cattle, sheep, goats or horses usually within 25 m, 2) forest and naturally created water pools, streams or swamps in neighbourhood, 3) electricity available, and 4) agreement of the farmer to operate the traps. The traps were placed outdoors at weatherproof sites, ideally on the stable wall at a height between 1.5 and 2.0 m above the ground, and with no strong light sources in the immediate vicinity. The insects were collected into 200 ml bottles containing 70% ethanol and which were sent to our institute every fortnight. The monitoring was carried out from June 2008 to May 2011 (total 156 weeks).

### Insect identification

#### Morphology

In a first step, *Culicoides* specimens were separated from the other insects using their characteristic features of wings, antennae, and legs. In a second step, females only were separated based on wing morphology into the Obsoletus group (*C. chiopterus*, *C. obsoletus*, *C. scoticus* and *C. dewulfi*, the latter phylogenetically not belonging to this group [30]), the Pulicaris group (*C. deltus*, *C. grisescens*, *C. impunctatus*, *C. lupicaris*, *C. newsteadi*, *C. pulicaris*, *C. punctatus*) and other *Culicoides* species. From catches that were estimated to contain more than 500 *Culicoides*, subsamples were analysed as described [31].

#### MALDI-TOF MS

MALDI-TOF MS-based identification of biting midges was carried out as described [27]. Briefly, the abdomens of the *Culicoides* specimens were removed, the remaining parts air-dried for approx. 2–4 min and individually transferred to 1.5 ml Eppendorf tubes where they were triturated in 10 μl of 25% formic acid using a manual homogenizer (Bio Vortexer, Fisher Scientific, Wohlen, Switzerland) with disposable pellet pestles. One μl of the homogenate was spotted in duplicate onto a steel target plate, air dried at room temperature for approx. 15 min, and 1 μl of SA matrix (saturated solution of sinapic acid in 60% acetonitrile, 40% H_2_O, 0.3% trifluoroacetic acid; all chemicals from Sigma-Aldrich, Buchs, Switzerland) was added directly onto the spots. After air-drying for 15 min, the plates were sent by overnight courier to a commercial company (Mabritec SA, Riehen, Switzerland) where the mass fingerprints were generated as described [27] and subjected to automated identification against >4,000 validated biomarker mass sets, including 15 *Culicoides* species-specific ones (Obsoletus group: *C. chiopterus*, *C. dewulfi*, *C. obsoletus*, *C. scoticus*; Pulicaris group: *C. deltus*, *C. grisescens* I, *C. grisescens* II {cryptic species [22]}, *C. lupicaris*, *C. pulicaris*, *C. punctatus*; and other *Culicoides*: *C. circumscriptus*, *C. festivipennis*, *C. imicola*, *C. nubeculosus*, *C. pallidicornis*) or, in case of no or low (<90%) identification value, to full spectra comparison against all available spectra (currently approx. 60,000 including all insect reference whole spectra).

#### PCR/sequencing

DNA was isolated from the retained abdomens with a kit (QIAamp DNA Mini Kit, Qiagen, Hilden, Germany) and the species determined by PCR/sequencing as described [22], sequencing by Synergene GmbH (Schlieren, Switzerland).

#### Statistics

Kruskal-Wallis tests (PASW Statistics 18 of SPSS) were used to detect temporal differences in the trapping success for all *Culicoides* and for the different *Culicoides* groups. Levels of significance (p<0.05) are given without and with Bonferroni correction to correct for multiple testing [32].

## Results

*Culicoides* biting midges were monitored at 12 locations in Switzerland over three years by overnight catching once a week. A total of nearly 550,000 biting midges were collected, together with around 1.3 million other insects as by-catch that entered the Onderstepoort blacklight traps (Table 1). The biting midges were separated by morphology into Obsoletus group, Pulicaris group and other *Culicoides*, with the first group dominating the Swiss midge fauna up to an altitude of 1,200 m, i.e. accounting for more than 80% of the midges. Farther up, the species of the Pulicaris group prevailed (85% of the biting midges at the highest trapping site at 2,130 m). Notable abundances of other *Culicoides* species were only observed at the single trapping site south of the Alps as well as at the three sites of high (>1,500 m) altitudes (Table 1).

The maximum number of midges collected in a single summer night largely varied among the sites, ranging from 756 (Commugny, altitude 420 m, trapping date 4 June 2008) to 19,682 (Dittigen, 360 m, 11 June 2008). Interestingly, the two highest altitude sites ranked second (n=18,129, Sils, 1,800 m, 02 August 2010) and fourth (n=9,633, Juf, 2,130 m, 12 August 2009) (not shown). Also, the overall number of midges collected over the tree years strongly varied at the different locations (from 6,933 to 149,439; Table 1).

**Figure 1:**
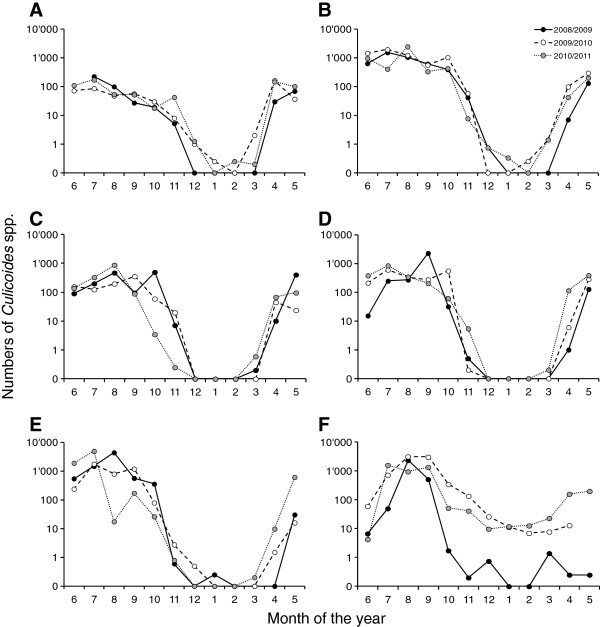
**Seasonal dynamics of biting midges at six trapping sites.** Data from the locations with high overall sampling reliability, i.e. at least 145 of the maximal 156 weekly catches available; see Table 1: **A**) Commugny (altitude 420 m); **B**) Wädenswil (620 m); **C**) Mühlethurnen (670 m); **D**) Chateau d’Oex (940 m); **E**) Chaumont (1,110 m); **F**) Juf (2,130 m). The monthly averages of total number of collected *Culicoides* biting midges are shown by black (season 2008/2009), open (2009/2010) and grey (2010/2011) circles. Note the logarithmic scale.

For further analyses, only the six trapping locations with high sampling reliability (i.e. at least 145 of the maximal 156 weekly catches available) were considered (see farms highlighted in grey in Table 1). The seasonal dynamics of the biting midges at these six sites over the three investigated years (June to May) is shown in Figure 1, displaying the monthly averages of the catches. Significant activity (more than 10 midges per trap and night) was observed between April and November, and on average less than one midge was collected during the winter months (December to March) with the exception of the location ‘Juf’ where a low abundance of around 10 midges were observed during the second and third trapping year (Figure 1F).

The variability of midge abundance (total *Culicoides* as well as the three different *Culicoides* groups separately) at the sites between the 3 years was analysed with the catches from June to September, which account for more than 80% of the annual midge collection in the vast majority of cases (Table 2). Statistically significant annual variation in midge abundance was neither found for the total number of midges nor for the majority of analyses of the midges belonging to the three groups (Table 2). Statistically significant variability between the years was determined in three instances (sites ‘Wädenswil’ and ‘Mühlethurnen’ for the Pulicaris group midges, site ‘Château d’Oex’ for the other *Culicoides* group, Table 2), all relating to midge groups with low abundances at these locations. Furthermore, only the variation of the numerically marginal Pulicaris group from ‘Mühlethurnen’ over the 3 years remained significant after Bonferroni correction for multiple testing (Table 2).

**Table 2:** **Statistical comparison**^**1**^**of the number of *Culicoides *collected at six locations**^**2**^

**Site (municipality)**	**Year (no. of trappings)**	**Total no. *Culicoides *(%**^**3**^**) *p-value***	**No.*Culicoides *of morphological group (%**^**3**^**)**		
			**Obsoletus**	**Pulicaris**	**Other *Culicoides***
			***p-value***	***p-value***	***p-value***
Commugny	2008 (14)	1,440 (75.4)	1,234 (73.4)	159 (91.4)	47 (85.3)
	2009 (18)	1,130 (55.9)	1,079 (55.5)	15 (68.2)	36 (64.3)
	2010 (19)	1,751 (58.8)	1,704 (58.3)	10 (66.7)	37 (97.4)
		*0.622*	*0.564*	*0.102*	*0.969*
Wädenswil	2008 (19)	16,603 (88.7)	15,022 (88.1)	1,215 (94.3)	366 (100.0)
	2009 (17)	23,306 (91.7)	22,387 (91.8)	564 (87.3)	355 (98.6)
	2010 (19)	19,351 (96.1)	18,495 (96.1)	576 (93.8)	280 (97.2)
		*0.588*	*0.574*	0.040^*^	*0.663*
Mühlethurnen	2008 (18)	4,179 (58.3)	4,011 (59.5)	98 (30.3)	70 (73.7)
	2009 (17)	3,712 (87.9)	3,617 (88.3)	51 (82.3)	44 (66.7)
	2010 (19)	6,015 (90.0)	5,907 (89.9)	18 (81.8)	90 (96.8)
		*0.889*	*0.857*	<0.008^*†^	*0.825*
Château	2008 (18)	13,516 (95.7)	11,991 (95.7)	1,350 (96.3)	175 (95.1)
d’Oex	2009 (16)	7,177 (81.9)	5,269 (82.3)	1,390 (87.3)	518 (67.7)
	2010 (19)	7,655 (77.2)	6,333 (75.3)	937 (83.7)	385 (99.5)
		*0.241*	*0.260*	*0.390*	0.018^*^
Chaumont	2008 (19)	28,859 (94.9)	21,095 (93.5)	7,601 (99.0)	163 (99.4)
	2009 (18)	16,725 (98.8)	12,596 (98.5)	3,951 (99.8)	178 (100.0)
	2010 (19)	30,076 (92.3)	28,090 (93.5)	1,840 (77.0)	146 (100.0)
		*0.763*	*0.709*	*0.311*	*0.931*
Juf	2008 (16)	12,215 (99.9)	165 (99.4)	11,158 (99.9)	892 (99.9)
	2009 (19)	31,635 (94.4)	780 (90.6)	26,026 (93.7)	4,829 (98.8)
	2010 (18)	17,145 (89.7)	203 (83.2)	15,006 (89.8)	1,936 (90.0)
		*0.336*	*0.270*	*0.466*	*0.069*

^1^ Kruskal-Wallis test; catches from the summer months (June to September) during the 3 years monitoring.

^2^ Locations with high overall sampling reliability (i.e. at least 145 of the maximal 156 weekly catches available, see Table 1).

^3^ Percentage of total *Culicoides* caught over the whole collecting season (June – May).

^*^ Significantly different using the Kruskal-Wallis test without Bonferroni correction (p < 0.05).

^*†*^ Significantly different using the Kruskal-Wallis test with Bonferroni correction (p < 0.008).

One hundred randomly selected individual biting midges per trapping site, all collected in late summer 2010 (period of highest abundance) and stored in 70% ethanol for more than 1 year, were subjected to MALDI-TOF MS analyses (Table 3). Thus, high quality spectra were obtained for 1,187 of the 1,200 (98.9%) analysed specimens of which 1,173 could be assigned to one of the 15 *Culicoides* species for which biomarker mass sets were available in the reference database. PCR/sequencing was done with six randomly chosen ( http://www.random.org) specimens per site, confirming in all cases the MALDI-TOF MS result (*C. obsoletus*, N=38; *C. grisescens* II, N=13; *C. scoticus*, N=11; *C. grisescens* I, N=5, *C. dewulfi*, N=2; 1 each *C. circumscriptus*, *C. deltus*, *C. lupicaris*, Table 3). Fourteen specimens yielded no MALDI-TOF MS identification, i.e. no corresponding reference spectra have as yet been deposited in the database. These 14 insects were identified by PCR/sequencing as *C. fascipennis* (n = 5), *C. reconditus* (3), *C. kibunensis* (2), *C. segnis*-like (2) and 1 each of *C. newsteadi* and *C. segnis*. Finally, thirteen insects (1.1%) could not be identified by MALDI-TOF MS due to low quality spectra (low signal/noise ratio, few masses due to signal suppression).

**Table 3:** **MALDI-TOF MS based species identification of 100 randomly selected *Culicoides *specimens from the 12 trapping sites**^**1**^

**Site**	***Culicoides *species (no.) {no. confirmed by PCR}**^**3 **^**[%**^**4**^**]**				
**(altitude**^**2**^**)**	**Morphological group**			**Unknown Genetic identification**^**4**^	**No identification**^**5**^
	**Obsoletus**	**Pulicaris**	**Other*Culicoides***		
Novazzano	*C. obsoletus* (84) {5}	*C. grisescens* II (1)	*C. circumscriptus* (4) {1}	0	0
(270)	*C. scoticus* (5)	*C. pulicaris* (1)	*C. pallidicornis* (4)		
		*C. punctatus* (1)			
	[91.4]	[1.9]	[6.7]		
Dittingen	*C. obsoletus* (3)	*C. lupicaris* (2)	0	0	0
(360)	*C. scoticus* (95) {6}				
	[99.9]	[0.1]	[0]		
Commugny	*C. obsoletus* (91) {6}	0	0	*C. kibunensis* (2)	3
(420)	*C. scoticus* (4)				
	[98.4]	[0]	[1.6]		
Granges	*C. obsoletus* (88) {6}	*C. grisescens* II (1)	0	0	0
(500)	*C. scoticus* (9)	*C. pulicaris* (2)			
	[100]	[0]	[0]		
Wädenswil	*C. obsoletus* (91) {5}	*C. lupicaris* (5) {1}	*C. pallidicornis* (1)	0	0
(620)	*C. scoticus* (2)	*C. pulicaris* (1)			
	[95]	[4.2]	[0.8]		
Mühlethurnen	*C. obsoletus*(74) {4}	*C. lupicaris* (1)	0	*C. segnis* (1)	3
(670)	*C. scoticus* (19) {1}				
	*C. dewulfi* (2) {1}				
	[99.0]	[1.0]	[0]		
Bennau	*C. obsoletus* (74) {4}	*C. grisescens* II (1) {1}	*C. pallidicornis* (1)	0	4
(810)	*C. scoticus* (11) {1}	*C. lupicaris* (9)			
	[92.1]	[5.6]	[2.2]		
Château d’Oex	*C. obsoletus* (91) {6}	*C. lupicaris* (1)	0	*C. newsteadi* (1)	0
(940)	*C. scoticus* (3)	*C. pulicaris* (3)			
		*C. punctatus* (1)			
	[90.6]	[4.7]	[4.7]		
Chaumont	*C. obsoletus* (12) {2}	*C. lupicaris* (1)	0	0	3
(1,110)	*C. scoticus* (75) {3}	*C. pulicaris* (1)			
	*C. chiopterus* (6)	*C. punctatus* (1)			
	*C. dewulfi* (1) {1}				
	[94.2]	[1.9]	[3.9]		
Davos	*C. obsoletus* (5)	*C. grisescens* I (4) {1}	*C. pallidicornis* (1)	0	0
(1,560)	*C. scoticus* (2)	*C. grisescens* II (87) {5}			
		*C. pulicaris* (1)			
	[4.2]	[95.0]	[0.8]		
Sils	*C. obsoletus* (4)	*C. deltus* (6) {1}	0	*C. fascipennis* (2)	0
(1,800)	*C. scoticus* (3)	*C. grisescens* I (5)		*C. reconditus* (1)	
		*C. grisescens* II (75) {5}			
		*C. pulicaris* (4)			
	[4.5]	[82.9]	[12.6]		
Juf		*C. grisescens* I (51) {4}	0	*C. fascipennis* (3)	0
(2,130)		*C. grisescens* II (40) {2}		*C. reconditus* (2)	
		*C. pulicaris* (2)		*C. segnis*-like (2)^6^	
	[1.5]	[94]	[4.5]		

^1^ Specimens randomly selected from a single catch from the high abundance season (August/September 2010), stored in 70% ethanol for more than 1 year and analysed using a database containing validated biomarker mass sets of 15 *Culicoides* species [27].

^2^ Meters above sea level.

^3^ PCR/sequencing [22] with 6 randomly chosen ( http://www.random.org) specimens/site. Species identification by comparison with GenBank entries (>99% identity).

^4^ Percentage of *Culicoides* morphologically classified as belonging to Obsoletus group, Pulicaris group or other *Culicoides* of the catch from which the specimens were selected for the MALDI-TOF MS analyses.

^4^ Identified by PCR/sequencing [22].

^5^ No species identification achievable due to poor quality mass spectra.

^6^ Best match: 96% identity (403/422) with GenBank entry HQ824509 (*Ccxx segnis* from Switzerland).

The MALDI-TOF-MS-based identification corresponded well with the rough morphological classification into the three groups Obsoletus, Pulicaris, other *Culicoides*. For example, the corresponding percentage of insects belonging to these groups as identified morphologically or by mass spectrometry were 91.4/89, 1.9/3 and 6.7/8, respectively, for the sample from the location ‘Novazzano’ (Table 3). This confirms the reliability of morphological identification to the group level when done by experienced personnel.

## Discussion

The spatio-temporal occurrence of *Culicoides* biting midges was determined in Switzerland over 3 years at 12 locations with one trap per site, at altitudes from 270 to 2,130 m. The six trapping locations (up to 670 m; Table 1) were part of a national monitoring system that included an additional 12 trapping sites in Switzerland and one in Liechtenstein up to a maximal altitude of 870 m. These 13 traps were only run from October until May to determine the vector-free period (i.e. less than five parous midges per trap night [33]) which is of high relevance when restriction measures for the movement of livestock are applied during an outbreak. Thus, for these two countries, the vector-free period lasts from week 47–49 to week 12–14, depending on weather conditions (for vector-free periods in other European countries see [6]). In order to also identify putatively 0vector-free areas, which are conceivable as retreat areas for animals during outbreaks of midge-borne diseases, six traps were placed at locations not covered by the national monitoring, particularly at higher altitudes. Monitoring activities in other European countries focussed on locations below 1,400 m (highest altitudes investigated: 1.400 m in Sicily [34]; 1,190 m in Austria [3]; 1,184 m in Central Italy [35]). The highest record of *Culicoides* in Switzerland was 1,600 m (from a zoological study [36]). We determined high abundances at the high altitude locations (1,800, 2,130 m; Table 1). In accordance with earlier preliminary studies [37,38], midges of the Pulicaris group consistently prevailed at high altitudes, as is the case at high latitudes in Scandinavia [5,39]. Nevertheless, biting midges of the Pulicaris group occasionally (single farms, few catches) occurred in higher numbers than the largely dominating Obsoletus group species in Central Europe [40,41]. However, in only one of our total 1,202 catches from the nine locations below 1,110 m altitude (Table 1) were the Pulicaris group species the most abundant (not shown). Species identification of 100 randomly selected midges per locations (by MALDI-TOF MS, see below) revealed a vast predominance of *C. obsoletus* (total 67.6%) and *C. scoticus* (24.8%) at the nine low altitude locations and of the cryptic species *C. grisescens* II (67.3%) at the three sites above 1,500 m. Highly interestingly, single specimens of *C. obsoletus* and *C. scoticus* were identified in the Alpine region and, vice versa, of *C. grisescens* II in the lowlands (Table 3), putatively due to the (albeit rare) long range dispersal of these tiny insects [42] and references cited therein. Further, whereas *C. obsoletus* was dominating in eight of the nine lower altitude locations, *C. scoticus* accounted for 95 of the 100 midges at the ninth location (‘Dittingen’, Table 3). As these analyses were done with insects from a single catch per location, obtained in summer, further analyses are required to understand the spatio-temporal population dynamics of the various species.

We observed a considerable range of the total number of collected biting midges between the locations. Such a variability between sites/farms is well known from many other studies e.g. [20,38,43-45]. In a previous study in Switzerland, for example, the total number of biting midges collected over one season at two farms only 4 km apart and located at the same altitude differed by the factor 24 [38]. Major factors influencing the abundance of midges are particularly topoclimate, land use and soil (as proxy for larval breeding sites) [35,46-48]. However, when comparing the number of midges we collected at the locations over three consecutive summers (June – September), statistically significant differences were neither observed for the total number nor for the vast majority of analyses of the three *Culicoides* groups (Table 2). Thus, collection of midges over the four summer months during one single year sufficed to obtain a representative picture of their abundances at a given location.

Our study was running over 3 years, adding up to a maximum total number of 156 weekly trappings per location. At six of the 12 locations, more than 145 catches were made (Table 1), and the data from these locations were used to depict the seasonal dynamics (Figure 1) and to analyse yearly differences in abundance (Table 2). The lower number of trappings at the other locations was due to technical problems (unnoticed failure of light bulbs/ventilator); missing/incorrect labelling of catches; independent decisions taken by farmers to skip trapping during adverse (mainly cold) weather conditions, and by simply forgetting to operate the traps by the farmers. A somewhat surprising and unique picture was obtained from the highest altitude trapping location (2,130 m) where, throughout two winters low midge activity was observed (Figure 1). We speculate that midges from the inside population escaped through shakes in the wooden wall of this barn. Such indoor populations of biting midges can reach considerable sizes [49-52] albeit these populations are also strongly reduced in winter [50] and own unpublished results from another trapping location.

MALDI-TOF MS has come of age for high throughput, accurate and reproducible identification of medically relevant microorganisms (bacteria, yeasts, filamentous fungi) at low costs and minimal preparation time [53]. Only recently, this proteomic approach has become available for *Culicoides* identification, relying on a validated reference database of biomarker mass sets from 15 *Culicoides* species [27], all but one (*C. imicola*) being indigenous to Switzerland. Analyses of 1,200 biting midges with MALDI-TOF MS confirmed the method’s reliability, as 98.9% of the specimens yielded high quality spectra, and 97.8% of the midges could be assigned to one of the species (Table 3) included in the database [27]. Obviously, the database covers the most abundant species of Central Europe. It is not clear how many indigenous *Culicoides* species exist in Switzerland. A compilation based on published data lists 35 established valid species [54]. In comparison, 51 species have been listed for north-eastern France, a region which has thoroughly been studied [18] (Delécolle, personal communication). However, several new species as well as specimens that could not unequivocally be identified by morphology have recently been reported from Switzerland [22,23,37,45]. In addition, cryptic species have been reported [21,22], and the genetic identification of two midges from the highest altitude trapping site as *C. segnis*-like (with 96% sequence identity to a *C. segnis* GenBank entry) indicates that the taxonomy of *Culicoides* midges remains an unfinished story.

No biomarker mass sets existed for 14 specimens, which therefore could not be assigned to a species. These 14 insects belonged to six species as identified by PCR/sequencing (Table 3), including five *C. fascipennis*. As the biomarker mass set for a species in general is derived from the reference spectra of at least five genetically confirmed specimens [27], a *C. fascipennis*-specific biomarker mass set comprising 29 masses (not shown) could be derived and added to the database.

Poor quality mass spectra, yielding no information with regard to species or group affiliation, were obtained for 13 (1.1%) specimens. The main source for this failure is most probably insufficient homogenization, which was done by a hand-held homogenizer, and automated sample preparation is desirable. With the already very high rate of good quality spectra (98.9%) obtained using a ‘quick and dirty’ preparation, it seems doubtful whether the evaluation of laborious refinements of pre-analytical processes might be a worthwhile expedient approach to further increase the efficiency of MALDI-TOF MS analyses.

Thus, MALDI-TOF MS is a new tool available for high throughput *Culicoides* species identification. This method is particularly economic for approaches requiring detailed and quantitative information on the midge fauna (to gain a rapid overview on the species present; to follow their spatio-temporal occurrence; to identify morphologically similar or indistinguishable species, e.g. *C. obsoletus* and *C. scoticus*) whereas PCR performed on DNA from pools of midges remains the method of choice for tracking down one or a few species of interest.

The foundation for MALDI-TOF MS analyses is the availability of a database with reference biomarker masses from carefully confirmed reference specimens. As the creation and maintenance of such a database is a tedious task, a centralised structure seems to offer an efficient solution. The database we rely on was created in collaboration between our group and a private company (Mabritec SA, Riehen, Switzerland) and, as shown in this work, enlarges when being utilized. Thus, this database might be of value as the core of an eventual comprehensive *Culicoides* database.

## Conclusion

There are no biting midge-free zones in the agriculturally utilized areas (including alpine summer pastures) of Switzerland, with midges of the Obsoletus group, which are considered the main vectors of bluetongue virus, dominating up to altitudes as high as 1,200 m. Above 1,500 m, the Pulicaris group whose species’ vector competences are largely unknown prevails with high populations. Our three-year monitoring revealed highly different numbers of *Culicoides* spp. collected at the 12 sites, but annual variations at the various locations were statistically insignificant, indicating that monitoring of midges preferably is done by investigating large numbers of sites for one season instead of few locations during extended periods of time. High throughput species identification of midges by MALDI-TOF MS is feasible. This technique adds to other recently developed methods for the identification of midges (PCRs in various formats, interactive identification keys), facilitating epidemiological and biological in-depth studies of these important insects.

## Competing interests

The authors declare that they have no competing interests.

## Authors’ contributions

AM and FS conceived the study; FS supervised the morphological and AM the genetical identification; CK and IS supervised and carried out the field and laboratory work; CK and DH analysed the data and interpreted the results; AM wrote the first draft of the paper, and all authors contributed to the final manuscript which they approve.
